# Predictive Values of Serum IL-33 and sST2 in Endotypes and Postoperative Recurrence of Chronic Rhinosinusitis with Nasal Polyps

**DOI:** 10.1155/2022/9155080

**Published:** 2022-05-19

**Authors:** Yanni Zhang, Kang Zhu, Jingguo Chen, Cui Xia, Chao Yu, Tianxi Gao, Jing Yan, Bingjie Zhao, Xiaoyong Ren

**Affiliations:** Department of Otolaryngology-Head and Neck Surgery, The Second Affiliated Hospital of Xi'an Jiaotong University, Xi'an, Shaanxi, China 710004

## Abstract

**Background:**

Chronic rhinosinusitis with nasal polyps (CRSwNP) is a common inflammatory disease with high heterogeneity and postoperative recidivation. The IL-33/ST2 axis is known to be involved in Th2 immune responses. This study is aimed at exploring levels of serum IL-33 and soluble ST2 (sST2) in CRSwNP patients and their potential for predicting CRSwNP endotypes and postoperative recurrence.

**Methods:**

The present study recruited 149 CRSwNP patients, 80 of whom were noneosinophilic (neCRSwNP) and 69 eosinophilic (eCRSwNP), as well as 60 healthy controls (HCs). Serum samples were collected from all participants, and sST2 and IL-33 concentrations were measured using ELISA. Multivariate analysis, receiver operating characteristic (ROC) curves, and Kaplan-Meier curves were used to evaluate the value of serum sST2 and IL-33 levels in distinguishing CRSwNP endotypes and predicting postoperative recurrence.

**Results:**

The levels of serum sST2 and IL-33 in CRSwNP patients were significantly higher than those in HCs, especially in the eCRSwNP group. Increased sST2 and IL-33 levels were associated with eosinophil counts and percentages in both tissue and blood. Multivariate regression and ROC curve analysis showed that serum sST2 and IL-33 exhibited potential for distinguishing CRSwNP endotypes, and the combination of serum IL-33 and sST2 showed even more predictive power. Finally, 124 CRSwNP patients completed the entire 3-year follow-up. Multivariate analysis and Kaplan-Meier curves showed that serum sST2 and IL-33 levels were associated with recurrence; serum sST2 and IL-33 each exhibited potential for predicting postoperative recurrence, and combining serum sST2 and IL-33 exhibited better accuracy and practicability.

**Conclusion:**

Our results suggested that serum sST2 and IL-33 levels were upregulated in CRSwNP patients and related to the degree of mucosal eosinophil infiltration and postoperative recurrence. Serum sST2 and IL-33 might serve as objective biomarkers for distinguishing phenotypes and predicting recurrence in CRSwNP, and their combined use outperformed either marker alone.

## 1. Introduction

Chronic rhinosinusitis (CRS) is a common disease characterized by chronic sinonasal mucosal inflammation [1]. Recent epidemiological studies reported that CRS affected approximately 5.5–28% of the global population, and the prevalence continues to increase [2–4]. Based on the presence of nasal polyps, CRS is grouped into CRS with nasal polyps (CRSwNP) and CRS without nasal polyps (CRSsNP) [5]. CRSwNP with dominant T helper 2 (Th2) inflammation can be further categorized into eosinophilic (eCRSwNP) and noneosinophilic (neCRSwNP) types determined by the degree of eosinophil infiltration in the nasal mucosa. These two endotypes have distinctly different clinical features, treatments, and prognoses [6]. Compared with neCRSwNP, patients with eCRSwNP tend to have more severe clinical symptoms, worse response to conventional treatment, and higher risk of recurrence [7, 8]. Therefore, preoperatively discriminating eCRSwNP from neCRSwNP and predicting postoperative recurrence are extremely important for rhinologists. It is essential to develop an objective biomarker to distinguish CRSwNP endotypes and predict recurrence prior to surgery.

Suppressor of tumorigenicity 2 (ST2) is a type 1 transmembrane protein encoded by the IL-1RL1 gene, which functions as an IL-33 receptor [9]. It is well known that IL-33 can induce a Th2-type inflammatory response and play a crucial role in allergic inflammatory reactions [10]. Previous studies showed that ST2 and IL-33 played vital roles in the regulation of immune and inflammatory responses and were involved in the pathological mechanisms of several inflammatory diseases [11, 12]. Magro and colleagues observed that serum soluble ST2 (sST2) levels were elevated in ulcerative colitis and correlated positively with disease severity, and they posited that ST2 signaling might contribute to the therapeutic response of golimumab treatment [13]. Recent publications reported that increased serum sST2 levels could initiate and amplify Th2 inflammatory responses and aggravate disease activity in asthma and food allergy [14–16]. Moreover, Liao et al. [17] found that expression levels of IL-33 and sST2 were elevated in the tissue samples of CRSwNP patients. Therefore, we considered that sST2 and IL-33 might play essential roles in the pathophysiology of CRSwNP and may be associated with its histopathology and postoperative recurrence.

## 2. Materials and Methods

### 2.1. Participants and Settings

For this study, we recruited 149 CRSwNP patients (including 80 neCRSwNP and 60 eCRSwNP) from October 2018 to January 2019. CRSwNP was diagnosed according to the guidelines of the European Position Paper on Rhinosinusitis and Nasal Polyps 2012 [18]. The exclusion criteria were as follows: (1) age < 18 years or >75 years; (2) undergoing treatment with antibiotics, oral or systemic corticosteroids, immunotherapy, or antiallergic drug treatment 4 weeks before surgery; (3) other nasal or sinus diseases; (4) other inflammatory or autoimmune diseases; and (5) severe heart disease, kidney or other organ dysfunction, or pregnancy. All patients with CRSwNP received routine preoperative examinations, including blood tests, nasal endoscopy, computed tomography (CT) or magnetic resonance imaging (MRI), and electrocardiogram, then rated their nasal symptoms using the widely accepted visual analogue scale (VAS) as described previously [19]. Preoperative CT and nasal endoscopy scores were recorded by the Lund-Mackay and Lund-Kennedy score systems, respectively [20]. A total of 60 age- and gender-matched healthy volunteers without rhinitis, sinusitis, or inflammatory or autoimmune diseases were recruited as healthy controls (HCs).

### 2.2. Diagnosis of eCRSwNP and neCRSwNP

Nasal polyp tissues were collected from all patients during the operation, then soaked in 10% formalin, and embedded with paraffin wax. Eosinophil counts were observed by hematoxylin-eosin (HE) staining in 10 randomly selected fields by two independent observers who were blinded to the clinical data. eCRSwNP was diagnosed when the percentage of eosinophils in the nasal polyp tissue was higher than 10% of total inflammatory cells; otherwise, a diagnosis of neCRSwNP was given [21–23].

### 2.3. Serum Sample Collection and Measurement of sST2 and IL-33 Levels

Five milliliters of peripheral blood was collected from CRSwNP patients and HCs by vacuum blood collection tubes in the morning. Blood was centrifuged at 1,200 × g for 10 min at 4°C; then, the supernatant was collected and stored at −80°C. Serum samples were thawed on ice before using. Serum sST2 and IL-33 measurements were performed by enzyme-linked immunosorbent assay using commercial human sST2 and IL-33 ELISA kits (Multisciences, Hangzhou, China) according to the manufacturer's instructions.

### 2.4. Follow-Up and Recurrence Evaluation

All patients underwent functional endoscopic sinus surgery and received standard postoperative management as described previously, including nasal and/or systemic medication and nasal saline irrigation [24, 25]. Follow-up examinations were performed at 0.5, 1, 2, and 3 years after surgery. Recurrence was defined as occurring when CRSwNP symptoms and nasal polyps reappeared and lasted more than 1 week, despite management with standard intranasal corticosteroids [26, 27]. The recurrent patients underwent a second functional endoscopic sinus surgery.

### 2.5. Statistical Analysis

All statistical analyses were conducted using SPSS software version 22.0 (IBM, Chicago, IL, USA), and figures were plotted with GraphPad Prism 7.0 (GraphPad Software Inc., La Jolla, CA, USA). Continuous and categorical variables were displayed as mean ± standard deviation (SD) and number (%), respectively. Student's *t*-test or Mann–Whitney *U* test was used to assess differences between the groups. The chi-square test was performed on categorical variables. Spearman's correlation analysis was conducted to evaluate the correlation between clinical parameters and sST2 and IL-33 levels. Receiver operating characteristic (ROC) curves were used to estimate area under the curve (AUC), sensitivity, and specificity. Based on the cutoff value, relapse-free survival analyses were conducted with Kaplan-Meier curves to evaluate the potential risk factors for postoperative recurrence. Statistical significance was set at *P* < 0.05.

## 3. Results

### 3.1. Baseline Characteristics of Study Participants

Of the 149 CRSwNP patients recruited to this study, 124 completed the entire follow-up and provided clinical data, and 25 dropped out. The main demographic and clinical characteristics of all subjects are shown in Table 1. There was no statistical difference in age, sex, smoking status, or BMI between the CRSwNP and HC groups (*P* > 0.05). The rates of atopy, allergic rhinitis, asthma, and blood eosinophil (B-EOS) counts and percentages were higher in the CRSwNP group than in HCs (*P* < 0.05). As listed in Table 2, the rates of atopy, allergic rhinitis, asthma, B-EOS counts and percentages, and tissue eosinophil (T-EOS) counts and percentages were higher in the eCRSwNP group than in the neCRSwNP group (*P* < 0.05). No statistical difference was observed between the two CRSwNP groups for age, gender, rate of smoking, BMI, preoperative VAS score, Lund-Mackay score, or Lund-Kennedy score (*P* > 0.05).

### 3.2. sST2 and IL-33 Levels Are Correlated with Clinical Variables

**Figure 1:**
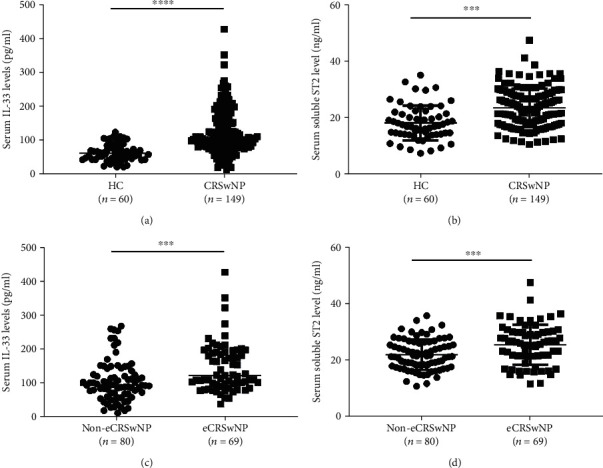
Serum IL-33 and sST2 concentrations were elevated in CRSwNP patients. (a) Serum IL-33 levels and (b) serum sST2 levels were significantly increased in the CRSwNP group as compared to HCs. Compared with the neCRSwNP group, (c) serum IL-33 and (d) serum sST2 levels were significantly higher in the eCRSwNP group. ^∗∗∗^*P* < 0.001; ^∗∗∗∗^*P* < 0.0001. IL-33: interleukin-33; sST2: soluble suppressor of tumorigenicity 2; CRSwNP: chronic rhinosinusitis with nasal polyps; HC: healthy control; neCRSwNP: noneosinophilic chronic rhinosinusitis with nasal polyps; eCRSwNP: eosinophilic chronic rhinosinusitis with nasal polyps.

Serum sST2 and IL-33 levels were significantly elevated in the CRSwNP group as compared with HCs (Figures 1(a) and 1(b)). Moreover, serum sST2 and IL-33 levels were higher in the eCRSwNP group than in the neCRSwNP group (Figures 1(c) and 1(d)). The Spearman correlation analysis showed that in CRSwNP patients, both sera sST2 and IL-33 exhibited positive associations with B-EOS counts and percentage as well as with T-EOS counts and percentage (Table 3).

### 3.3. sST2 and IL-33 Levels as Potential Biomarkers for Predicting eCRSwNP

**Figure 2:**
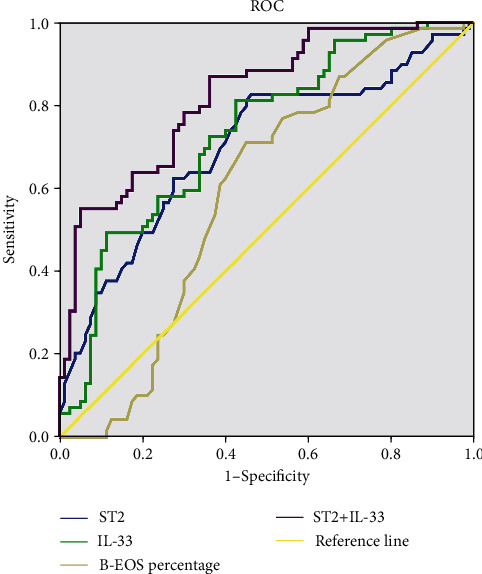
ROC curves of potential predictive variables for distinguishing CRSwNP endotypes. ROC: receiver operating characteristic; CRSwNP: chronic rhinosinusitis with nasal polyps; IL-33: interleukin-33; sST2: soluble suppressor of tumorigenicity 2; B-EOS: blood eosinophil.

In order to investigate potential factors associated with endotypes of CRSwNP, variables with significant differences were included in a binary logistic regression analysis (Table 4). Both adjusted and unadjusted results indicated that serum sST2 level, serum IL-33 level, B-EOS percentage, and asthma were associated with CRSwNP endotypes. ROC curves showed that serum sST2 and IL-33 offer potential for distinguishing CRSwNP endotypes, and combining serum sST2 and IL-33 exhibited even greater predictive value (Figure 2 and Table S1).

### 3.4. Serum sST2 and IL-33 Levels in Patients with Recurrent CRSwNP

**Figure 3:**
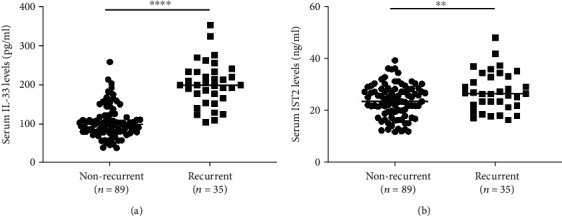
Serum IL-33 and sST2 concentrations were elevated in recurrent CRSwNP patients as compared to nonrecurrent patients. IL-33: interleukin-33; sST2: soluble suppressor of tumorigenicity 2; CRSwNP: chronic rhinosinusitis with nasal polyps; ^∗∗^*P* < 0.01; ^∗∗∗∗^*P* < 0.0001.

**Figure 4:**
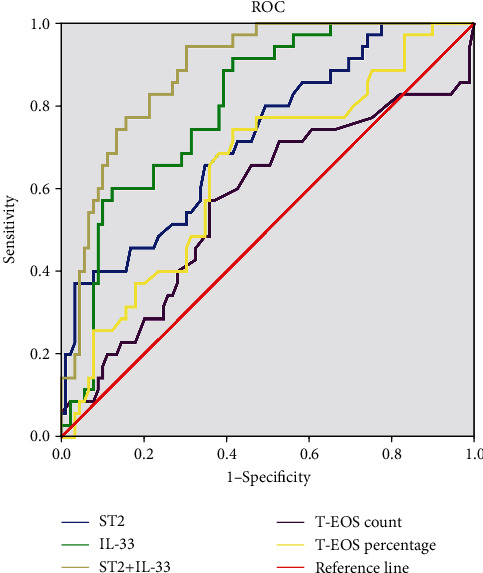
ROC curves of novel biomarkers for predicting postoperative recurrence in CRSwNP patients. ROC: receiver operating characteristic; CRSwNP: chronic rhinosinusitis with nasal polyps; IL-33: interleukin-33; sST2: soluble suppressor of tumorigenicity 2; T-EOS: tissue eosinophil.

**Figure 5:**
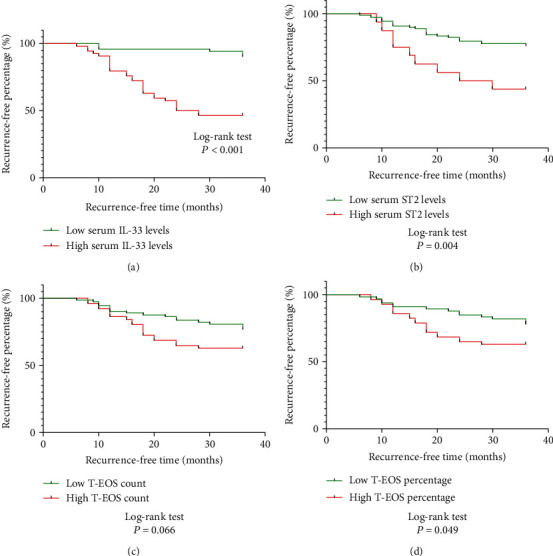
Kaplan-Meier plots of candidate biomarkers for predicting recurrence in CRSwNP patients. Serum IL-33 (a), sST2 (b), and T-EOS percentage (d) showed significant differences in predictive power (*P* < 0.05), but T-EOS count (c) did not. CRSwNP: chronic rhinosinusitis with nasal polyps; IL-33: interleukin-33; sST2: soluble suppressor of tumorigenicity 2; T-EOS: tissue eosinophil.

A comparison of demographic and clinical characteristics between the nonrecurrent and recurrent CRSwNP groups is shown in Table 5. Statistical differences were observed between the two groups for comorbid atopy, allergic rhinitis, asthma, B-EOS percentages, and T-EOS counts and percentages (*P* < 0.05). As shown in Figure 3, serum IL-33 and sST2 levels were significantly elevated in the recurrent group as compared to the nonrecurrent group (*P* < 0.05). Adjusted and unadjusted logistic regression analyses suggested that comorbid allergic rhinitis, B-EOS percentages, T-EOS counts and percentages, and serum sST2 and IL-33 levels were associated with postoperative recurrence (*P* < 0.05) (Table 6). The ROC analysis results in Figure 4 showed that serum sST2 and IL-33 exhibited potential for predicting postoperative recurrence, and combining serum sST2 and IL-33 exhibited better accuracy and practicability (*P* < 0.05). The detailed data from these analyses are presented in Table S2. Moreover, Kaplan-Meier plots demonstrated that serum sST2 and IL-33 exhibited significantly different postoperative recurrences (Figure 5).

## 4. Discussion

In the present study, we found that serum sST2 and IL-33 levels were significantly higher in CRSwNP patients than in HCs, and their levels were positively correlated with eosinophil counts and percentages in both tissue and blood. Moreover, binary logistic regression analysis and ROC curve analysis showed that serum sST2 and IL-33 were closely associated with CRSwNP endotypes. In addition, serum sST2 and IL-33 levels were significantly higher in recurrent CRSwNP patients than in nonrecurrent patients, and serum sST2 and IL-33 were both risk factors for postoperative recurrence. Given these findings, we suggest that serum sST2 and IL-33 might be crucial biomarkers for distinguishing CRSwNP endotypes and predicting recidivation. Notably, a combination of these two indicators showed even more predictive ability.

IL-33, as a proinflammatory factor, is involved in the development of chronic airway diseases through promoting Th2-type cytokine secretion and eosinophil infiltration [28]. ST2, the receptor of IL-33, is also a key component in promoting Th2 responses and triggering eosinophilic inflammation in allergic diseases [29, 30]. ST2 is expressed in a variety of immune cells, such as mast cells, macrophages, dendritic cells, and eosinophils, and plays a key role in regulating immune and inflammatory responses [31, 32]. Previous studies have shown that IL-33/ST2 signaling is involved in the occurrence and development of various inflammatory and allergic diseases, including rheumatoid arthritis, inflammatory bowel disease, food allergy, and asthma [33–35]. Huang et al. [35] observed that elevated ST2 could promote CD4+ T cells toward Th2 differentiation by activating myeloid dendritic cells. eCRSwNP is a Th2 cytokine-mediated chronic inflammatory disease, and tissue eosinophil infiltration is a major pathological feature [36]. Moreover, the positive feedback loop between IL-33/sST2 and Th2 cytokines may facilitate Th2-skewed inflammation in eCRSwNP [17]. In the present study, we demonstrated that serum sST2 and IL-33 concentrations were significantly higher in CRSwNP patients, especially in eCRSwNP patients, and the elevated sST2 and IL-33 levels were associated with tissue and circulating eosinophilic inflammation. Furthermore, ROC analysis showed that serum sST2 and IL-33 each exhibited predictive power in distinguishing CRSwNP endotypes and their combination exhibited even greater predictive value. Therefore, we have reason to believe that the IL-33/ST2 axis plays a pivotal role in the histopathology of CRSwNP. However, further experimental studies are needed to uncover the underlying mechanism.

Although current options for diagnosis and treatment (especially functional endoscopic sinus surgery) significantly improved the life quality for CRSwNP patients, the postoperative recurrence rate remained high, with a long-term surgical revision rate of 15–20% [37]. A recent study found that CRSwNP patients with increased eosinophil counts and Th2 cytokine levels in nasal biopsy specimens were more likely to suffer postoperative recurrence [38]. Therefore, exploring reliable indicators for predicting the recurrence of CRSwNP is urgently needed. Although several variables and indicators have been previously associated with CRSwNP recurrence, including concomitant asthma [39], peripheral parameters [24], and nasal microbiota [27], none of them are available in regular clinical practice. Here, we found that serum sST2 and IL-33 levels were significantly elevated in recurrent CRSwNP patients. Previous studies indicated that the degree of Th2 inflammation and eosinophil infiltration was a major predictor of CRSwNP recurrence [40, 41]. Indeed, in the present study, we found that B-EOS percentages and T-EOS counts and percentages were higher in CRSwNP patients and associated with postoperative recurrence [24]. We also observed that serum sST2 and IL-33 levels correlated positively with eosinophil counts and percentages in both blood and tissue, suggesting that serum sST2 and IL-33 aggravated the tissue and stimulated circulating eosinophilic inflammation. Previous studies demonstrated that IL33 and ST2 could promote Th2-type inflammation, and IL-33 could drive eosinophil infiltration into the nasal mucosa [42, 43]. Therefore, we presumed that high levels of circulating IL-33 and sST2 could promote Th2 activation and cytokine secretion and facilitate eosinophilic infiltration into the tissue, thus enhancing the risk of postoperative recurrence in CRSwNP patients. Together, our results suggest that serum IL-33 and sST2 levels can be used as objective biomarkers to predict postoperative recurrence in CRSwNP patients.

This study had some limitations. First, the number of patients recruited in this study is relatively small, and additional validation studies are needed to strengthen the conclusions. Second, all recruited participants came from a single medical center and were of the same race, so external validation from other, more diverse, institutions are needed to be able to generalize our findings. Finally, we did not evaluate serum ST2 and IL-33 levels between baseline and 3 years after surgery, which may limit the clinical application.

## 5. Conclusion

This prospective study observed that serum levels of sST2 and IL-33 were increased in CRSwNP patients and were associated with mucosal eosinophilia and postoperative recurrence. We also demonstrated that serum IL-33 and sST2 each have potential as biomarkers for distinguishing CRSwNP endotypes and predicting recurrence, and together, they exhibit even greater predictive power. The findings of this study contributed to understanding the underlying mechanisms of CRSwNP and improving precise treatment.

## Figures and Tables

**Table 1:** Demographic and clinical characteristics of all participants.

Variables	HC (*n* = 60)	CRSwNP (*n* = 149)	*P*
Age (years)	34.6 ± 7.3	36.2 ± 12.4	0.351
Male/female	32/28	82/67	0.878
Smoking, *n* (%)	13 (21.7)	36 (24.2)	0.857
BMI (kg/m^2^)	23.4 ± 1.8	23.7 ± 2.1	0.332
Atopy, *n* (%)	0 (0)	28 (18.8)	<0.001
Allergic rhinitis, *n* (%)	0 (0)	32 (21.5)	<0.001
Asthma, *n* (%)	0 (0)	18 (12.1)	<0.001
B-EOS counts (10^6^/L)	165.5 ± 37.2	259.4 ± 73.5	<0.001
B-EOS percentage (%)	1.9 ± 0.8	3.4 ± 1.3	<0.001

CRSwNP: chronic rhinosinusitis with nasal polyps; HC: healthy control; BMI: body mass index; B-EOS: blood eosinophil.

**Table 2:** Comparison of demographic and clinical characteristics between the neCRSwNP and eCRSwNP groups.

Variables	neCRSwNP (*n* = 80)	eCRSwNP (*n* = 69)	*P*
Age (years)	33.0 ± 12.4	34.5 ± 14.1	0.491
Male/female	45/35	37/32	0.869
Smoking, *n* (%)	20 (25.0)	16 (23.2)	0.849
BMI (kg/m^2^)	23.8 ± 2.2	23.5 ± 2.0	0.388
Atopy, *n* (%)	7 (7.2)	21 (30.4)	0.001
Allergic rhinitis, *n* (%)	10 (12.5)	22 (31.9)	0.005
Asthma, *n* (%)	5 (6.3)	13 (18.8)	0.023
B-EOS count (10^6^/L)	195.8 ± 58.5	342.6 ± 98.4	<0.001
B-EOS percentage (%)	2.6 ± 1.0	4.6 ± 2.1	<0.001
Lund-Mackay score	19 (15, 21)	18 (15, 20)	0.873
Lund-Kennedy score	9 (6, 11)	9 (7, 11)	0.921
VAS score	5 (3, 7)	5 (3, 6)	0.945
T-EOS count	14.2 (10.4, 19.1)	50.6 (36.8, 64.4)	<0.001
T-EOS percentage (%)	6.5 (3.6, 8.5)	21.1 (15.6, 28.3)	<0.001

neCRSwNP: noneosinophilic chronic rhinosinusitis with nasal polyps; eCRSwNP: eosinophilic chronic rhinosinusitis with nasal polyps; BMI: body mass index; B-EOS: blood eosinophil; VAS: visual analogue scale; T-EOS: tissue eosinophil.

**Table 3:** Association between serum IL-33 and ST2 levels and clinical parameters in CRSwNP patients.

Variable	Serum IL-33 level		Serum ST2 level	
	*r*	*P* value	*r*	*P* value
Age	-0.212	0.879	-0.187	0.792
BMI	0.102	0.549	0.092	0.568
B-EOS count (10^6^/L)	0.417	0.006	0.489	0.002
B-EOS percentage (%)	0.389	0.027	0.402	0.009
Lund-Mackay score	0.187	0.613	0.192	0.598
Lund-Kennedy score	0.078	0.914	0.067	0.896
VAS score	0.193	0.734	0.217	0.698
T-EOS count	0.612	<0.001	0.594	<0.001
T-EOS percentage (%)	0.656	<0.001	0.702	<0.001
Serum IL-33 level (pg/mL)	—	—	0.793	<0.001
Serum ST2 level (ng/mL)	0.793	<0.001	—	—

ST2: suppressor of tumorigenicity 2; CRSwNP: chronic rhinosinusitis with nasal polyps; BMI: body mass index; B-EOS: blood eosinophil; VAS: visual analogue scale; T-EOS: tissue eosinophil.

**Table 4:** Unadjusted and adjusted multivariate logistic regression analysis of factor associated with eCRSwNP. Resutls were adjusted for age, gender, smoking, BMI, atopy, allergic rhinitis, asthma and Lund-Mackay score, Lund-Kennedy score, and VAS score.

Factor	Unadjusted		Adjusted	
	OR (95% CI)	*P*	OR (95% CI)	*P*
Atopy	1.272 (0.985-2.765)	0.768	1.387 (0.913-2.986)	0.812
Allergic rhinitis	1.784 (1.152-3.869)	0.030	1.591 (0.938-3.024)	0.077
Asthma	2.053 (1.287-4.876)	0.042	1.867 (1.319-4.767)	0.016
B-EOS count (10^6^/L)	1.479 (1.176-2.954)	0.029	1.381 (0.897-2.482)	0.164
B-EOS percentage (%)	1.985 (1.358-5.897)	0.008	2.323 (1.786-6.056)	0.019
Serum IL-33 level (pg/mL)	2.871 (1.674-8.992)	0.005	2.732 (1.476-9.568)	0.001
Serum ST2 level (ng/mL)	2.439 (1.176-7.569)	<0.001	2.856 (1.285-8.789)	<0.001

eCRSwNP: eosinophilic chronic rhinosinusitis with nasal polyps; B-EOS: blood eosinophil; ST2: suppressor of tumorigenicity 2; OR: odds rate; CI: confidence interval.

**Table 5:** The demographic and clinical characteristics between the nonrecurrent and recurrent groups.

Variables	Nonrecurrent (*n* = 89)	Recurrent (*n* = 35)	*P*
Age (years)	33.1 ± 8.8	34.7 ± 8.5	0.361
Male/female	55/34	19/16	0.542
Smoking, *n* (%)	24 (27.0)	7 (20.0)	0.495
BMI (kg/m^2^)	23.8 ± 2.2	23.5 ± 2.0	0.388
Atopy, *n* (%)	8 (9.0)	11 (31.4)	0.004
Allergic rhinitis, *n* (%)	9 (10.1)	14 (40.0)	<0.001
Asthma, *n* (%)	5 (5.6)	8 (22.9)	0.009
B-EOS count (10^6^/L)	272.5 ± 119	296.9 ± 108.9	0.296
B-EOS percentage (%)	2.7 ± 1.2	3.5 ± 1.4	0.004
Lund-Mackay score	18 (14, 21)	19 (15, 22)	0.286
Lund-Kennedy score	8 (5, 11)	10 (8, 11)	0.102
VAS score	4 (3, 6)	5 (3, 7)	0.723
T-EOS count	14.2 (10.4, 19.1)	50.6 (36.8, 64.4)	<0.001
T-EOS percentage (%)	9.8 (6.7, 18.5)	23.2 (16.4, 32.1)	<0.001

BMI: body mass index; B-EOS: blood eosinophil; VAS: visual analogue scale; T-EOS: tissue eosinophil.

**Table 6:** Unadjusted and adjusted multivariate logistic regression analysis of risk factors associated with CRSwNP recurrence.

Factor	Unadjusted		Adjusted^∗^	
	OR (95% CI)	*P*	OR (95% CI)	*P*
Atopy	1.854 (0.916-4.956)	0.419	1.592 (0.897-4.045)	0.710
Allergic rhinitis	1.529 (1.067-6.187)	0.007	1.396 (1.034-6.287)	0.015
Asthma	1.793 (1.158-5.287)	0.038	1.669 (0.911-3.892)	0.092
B-EOS counts (10^6^/L)	1.378 (0.895-3.065)	0.387	1.258 (0.913-3.749)	0.198
B-EOS percentage (%)	2.162 (0.927-6.194)	0.467	1.867 (1.142-5.695)	0.025
T-EOS counts	2.367 (1.383-9.673)	0.002	2.198 (1.386-8.673)	0.009
T-EOS percentage (%)	2.980 (1.266-8.574)	<0.001	2.679 (1.197-9.266)	0.001
Serum IL-33 level (pg/mL)	3.769 (1.478-10.269)	<0.001	4.451 (1.313-11.672)	<0.001
Serum ST2 level (ng/mL)	3.687 (1.489-10.691)	<0.001	3.916 (1.195-12.764)	<0.001

CRSwNP: chronic rhinosinusitis with nasal polyps; B-EOS: blood eosinophil; ST2: suppressor of tumorigenicity 2; OR: odds rate; CI: confidence interval. ^∗^Adjusted for age, gender, smoking, BMI, atopy, allergic rhinitis, asthma, Lund-Mackay score, Lund-Kennedy score, and VAS score.

## Data Availability

The data used to support the observations of this study are available from the corresponding author upon request.

## Supplementary Materials

Supplementary Materials Table S1: the ROC analysis results of parameters associated with CRSwNP endotypes. Table S2: the ROC analysis results of parameters associated with CRSwNP recurrence.
