# Supplementary material for: Predictive Values of Serum IL-33 and sST2 in Endotypes and Postoperative Recurrence of Chronic Rhinosinusitis with Nasal Polyps
Source: Mediators Inflamm. 2022 May 19;2022:9155080. doi: 10.1155/2022/9155080 (PMC9135518; doi:10.1155/2022/9155080)
Supplement: Supplementary Materials — Table S1: the ROC analysis results of parameters associated with CRSwNP endotypes. Table S2: the ROC analysis results of parameters associated with CRSwNP recurrence. [file 9155080.f1.docx]

| Parameters | AUC (95% CI) | P | Cutoff | Sensitivity | Specificity |
| --- | --- | --- | --- | --- | --- |
| B-EOS percentage (%) | 0.592 (0.499-0.685) | 0.053 | 3.0 | 0.667 | 0.575 |
| Serum IL-33 level (pg/ml) | 0.733 (0.653-0.813) | <0.001 | 98.9 | 0.812 | 0.575 |
| Serum ST2 level (ng/ml) | 0.700 (0.614-0.786) | <0.001 | 25.1 | 0.623 | 0.725 |
| ST2+IL-33 | 0.823 (0.758-0.889) | <0.001 |  |  |  |

Table S1 ROC analysis of parameters associated with CRSwNP endotypes

ROC, receiver operating characteristics; CRSwNP, chronic rhinosinusitis with nasal polyps; B-EOS, blood eosinophil; ST2, suppressor of tumorigenicity 2; AUC, area under the curve; CI, confidence interval

Table S2 ROC analysis of parameters associated with CRSwNP recurrence

| Parameters | AUC (95% CI) | P | Cutoff | Sensitivity | Specificity |
| --- | --- | --- | --- | --- | --- |
| T-EOS count | 0.568 (0.449-0.687) | 0.240 | 30.5 | 0.571 | 0.640 |
| T-EOS percentage (%) | 0.645 (0.538-0.752) | 0.012 | 12.8 | 0.743 | 0.587 |
| Serum IL-33 level (pg/ml) | 0.799 (0.719-0.878) | <0.001 | 118.7 | 0.914 | 0.584 |
| Serum ST2 level (ng/ml) | 0.722 (0.623-0.820) | <0.001 | 32.1 | 0.371 | 0.966 |
| ST2+IL-33 | 0.883 (0.825-0.942) | <0.001 |  |  |  |

ROC, receiver operating characteristics; CRSwNP, chronic rhinosinusitis with nasal polyps; T-EOS, tissue eosinophil; ST2, suppressor of tumorigenicity 2; AUC, area under the curve; CI, confidence interval
